# The influence of sac centreline on saccular aneurysm rupture: computational study

**DOI:** 10.1038/s41598-023-38466-2

**Published:** 2023-07-12

**Authors:** Hao Jiang, Zhiwei Lu, M. Barzegar Gerdroodbary, Amir Sabernaeemi, Sajad Salavatidezfouli

**Affiliations:** 1 Dept. Neurosurg, Shulan (Hangzhou) Hospital Affiliated to Zhejiang Shuren University Shulan International Medical College, Hangzhou, 310000 Zhejiang China; 2 Hangzhou Heyunjia Hospital, Hangzhou, 310000 Zhejiang China; 3 Department of Mechanical Engineering, Babol Noshirvani University of Technology, Babol, Iran; 4 Department of Space, Earth and Environment, Chalmers University of Technology, Gothenburg, Sweden; 5 Mathematics Area, MathLab, International School for Advanced Studies (SISSA), Trieste, Italy

**Keywords:** Biomedical engineering, Mechanical engineering

## Abstract

The geometric characteristics of a saccular aneurysm play a crucial role in its rupturing. This article thoroughly investigates the impact of the sac centerline on aneurysm rupture, with a focus on identifying significant factors related to rupture at different time intervals. The study employs comprehensive computational simulations of six models of the ICA with varying coiling porosities and blood HCTs, using CFD analysis to examine WSS, OSI, pressure, and velocity within the saccular aneurysm for different sac centerlines. The results indicate that higher blood HCT levels lead to increased WSS and pressure values on the aneurysm wall, while OSI and mean velocity decrease. The study also reveals that coiling techniques can significantly reduce the risk of rupture, as decreasing coil porosity (increasing coil permeability) increases OSI and pressure while decreasing WSS and blood velocity within the aneurysm sac.

## Introduction

Intracranial aneurysms (IAs) refer to the central dilatation of vessel walls, which commonly occur along the branches of the Circle of Willis. These cerebrovascular disorders carry a high risk of mortality and morbidity upon aneurysm rupture, with approximately 45% of cases resulting in permanent disability or death and possible damage to surrounding cerebral tissues. The prevalence of IAs in the adult population is estimated to be around 5–9%. However, with advances in medical imaging technology over the last three decades, more than 50% of cerebral aneurysms are now detected before rupture occurs. This has provided researchers with access to valuable data and information about the geometric characteristics of aneurysms, which is essential for selecting appropriate treatments^1–3^.

Previous studies^4,5^ have made extensive efforts to highlight the significant role of CFD in the detection and treatment of this disorder. Most of these studies have confirmed the importance of wall shear stress in the initiation stage due to its impact on the endothelial layer ^6,7^. This layer is capable of converting mechanical signals into biological signals and activating biochemical pathways within the vessel wall.

The orientation and structure of the endothelial cells are altered when flow conditions deviated from normal status^8,9^. Consequently, a damaging remodeling of the vessel wall occurs over the above-mentioned events. The precise character of the hemodynamics is not completely preserved, but, extensively recognized research done by sadeh et al.^10^ indicates that an abnormal hemodynamical situation including high WSSG and WSS initiates the negative reorientation and reshaping of the endothelial monolayer and plane muscle cells^11,12^. Besides, this layer under disturbed flow conditions is known to exhibit several pathological features^13,14^.

Coiling embolism is a popular technique for treating saccular aneurysms^15,16^. The effectiveness of this method in reducing the risk of aneurysm rupture depends on its impact on the hemodynamics of different aneurysms^17,18^. By applying CFD to simulate blood flow within the ICA with coiling embolism, the hemodynamic aspects of coiling can be revealed, and the required percentage of coil for risk reduction of aneurysm rupture can be determined^14,16^. However, due to the complexity and diversity of aneurysm shapes and geometries, reliable research on this topic requires real 3-D geometries of aneurysms^5^. In this study, six different ICA shapes were chosen to investigate the effect of aneurysm shape on the efficiency of coiling. Blood flow analysis was also performed on these models to examine their hemodynamic characteristics.

This study aims to investigate the evolving flow characteristics within reassembled pre-aneurysmal shapes by analyzing local flow pathways. Computational fluid dynamics (CFD) is used to simulate blood flow within cerebral aneurysms. The study identifies a primary and secondary flow pathway based on the orientation of the sac and vessel centerlines. Six different internal carotid aneurysms (ICAs) are analyzed to determine the main effects of blood hematocrit and coiling embolism on wall shear stress (WSS) and oscillatory shear index (OSI) values.

## Material and methods

It is confirming that all methods were carried out in accordance with relevant guidelines and regulations. Besides, all experimental protocols were approved by of the Ca' Granda Niguarda Hospital and it is confirmed that informed consent was obtained from all subjects and/or their legal guardian(s).

**Figure 1:**
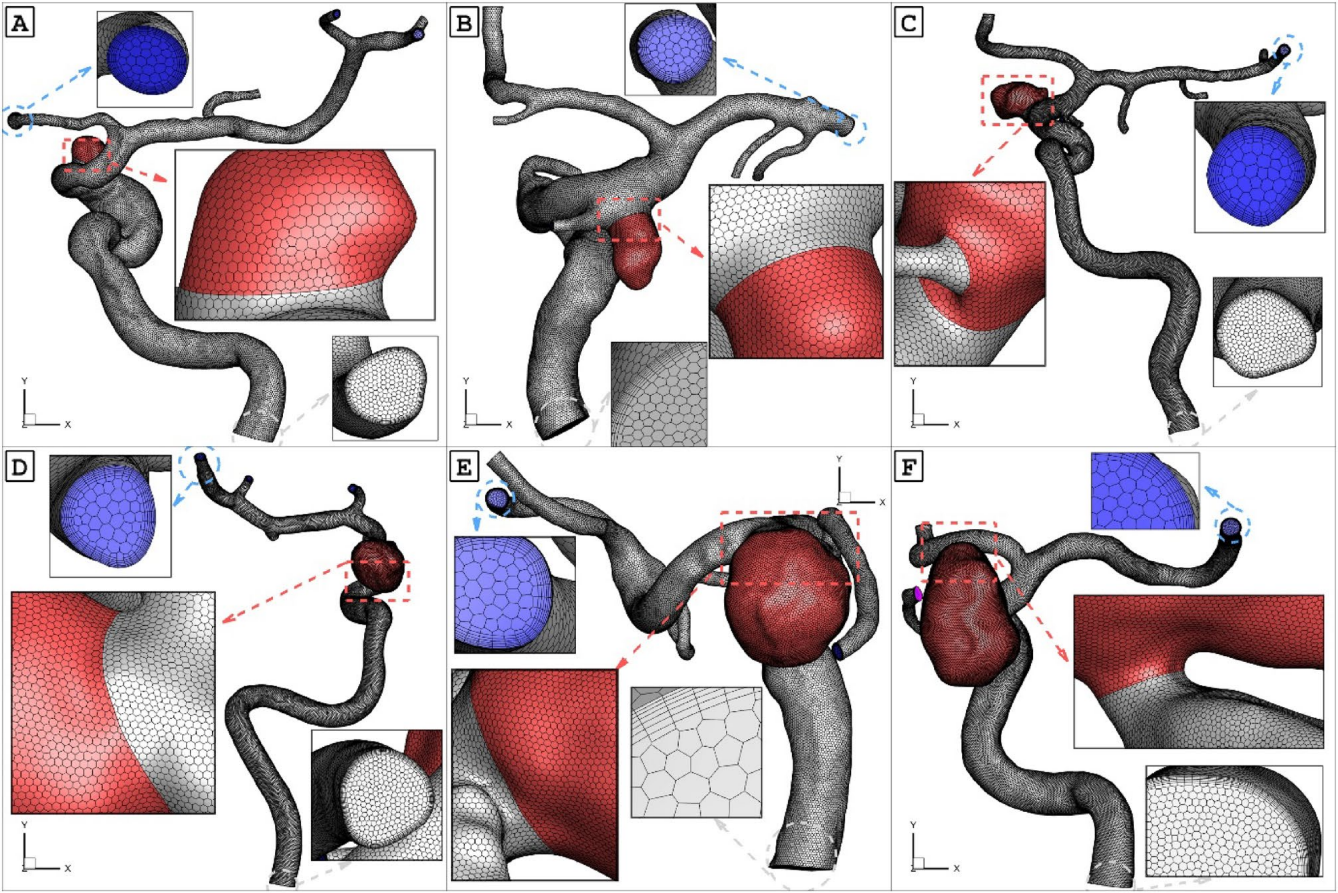
six cases with applied grids.

As previously mentioned, real 3-dimensional models of the ICA are crucial for investigating the impact of blood on aneurysm rupture when coiling embolism is applied. For this study, we selected six ICA models from Aneurisk^19^ for our simulations. Given the importance of this factor on aneurysm rupture, we aimed to examine and compare the distribution of wall shear stress (WSS), oscillatory shear index (OSI), and pressure on the six selected models. The geometric details of the aneurysms chosen for analysis are presented in Table 1, and their geometrical features are nearly identical (Fig. 1). All selected cases, except for Model B, are unruptured models.

**Table 1:** Details of selected ICA aneurysm.

Model	Sac Volume	Sac Centerline Length	WSS Min Sac (pa)	OSI Max Sac	Mean Wall Pressure (Pa)	Max Wall Pressure (Pa)	Mean Sac Pressure (Pa)	Max Sac Pressure (Pa)	OSI Mean Sac	Mean Vel Sac m/s	Max Vel Sac m/s	MVA_Int m/s
A	30.80887	3.478118	1.224985	0.162072	24502.37	24577.9	24486.24	24579.01	0.00588	0.188099	0.57415	0.279609
B	68.82005	5.762182	0.861677	0.207146	18061.55	18213.61	17987.76	18216.28	0.006905	0.43264	0.983588	0.697393
C	125.2596	6.510948	0.307939	0.31419	19191.12	19830.16	19132.63	19838.42	0.00776	0.366304	1.223228	
D	289.3767	8.308456	0.230072	0.375356	19423.67	20180.7	19328.86	20189.18	0.013998	0.509628	1.756638	0.661451
E	533.6227	10.65323	0.108216	0.383909	19213.4	19376.57	19194.86	19377.64	0.008466	0.173294	0.907829	0.429496
F	1312.026	15.31556	0.002808	0.478797	17,505.05	17,689.06	17,507.37	17,678.75	0.058676	0.026245	1.649474	0.396914

For the computational study of blood flow through cerebral aneurysms, the unsteady Navier–Stokes equations are typically solved, and blood flow is considered non-Newtonian. In this study, a commercial software called ANSYS, which is commonly used for engineering and biomedical modeling^20–25^, was used for CFD simulations. The carotid vessel walls and bloodstream were modeled along the axial direction using either fully coupled or one-way fluid–structure interaction (FSI) simulations. In the one-way FSI simulation, the fluid model was solved first, and the pressure obtained was transferred to the solid domain of the wall as an exterior loading for estimating the structural stress, without any additional interaction between the solid and fluid domains. In contrast, the fully coupled FSI simulation used non-linear incremental iterative procedures for interaction between the two domains. In this study, the one-way FSI procedure was applied for simulating blood flow inside the aneurysm.

**Figure 2:**
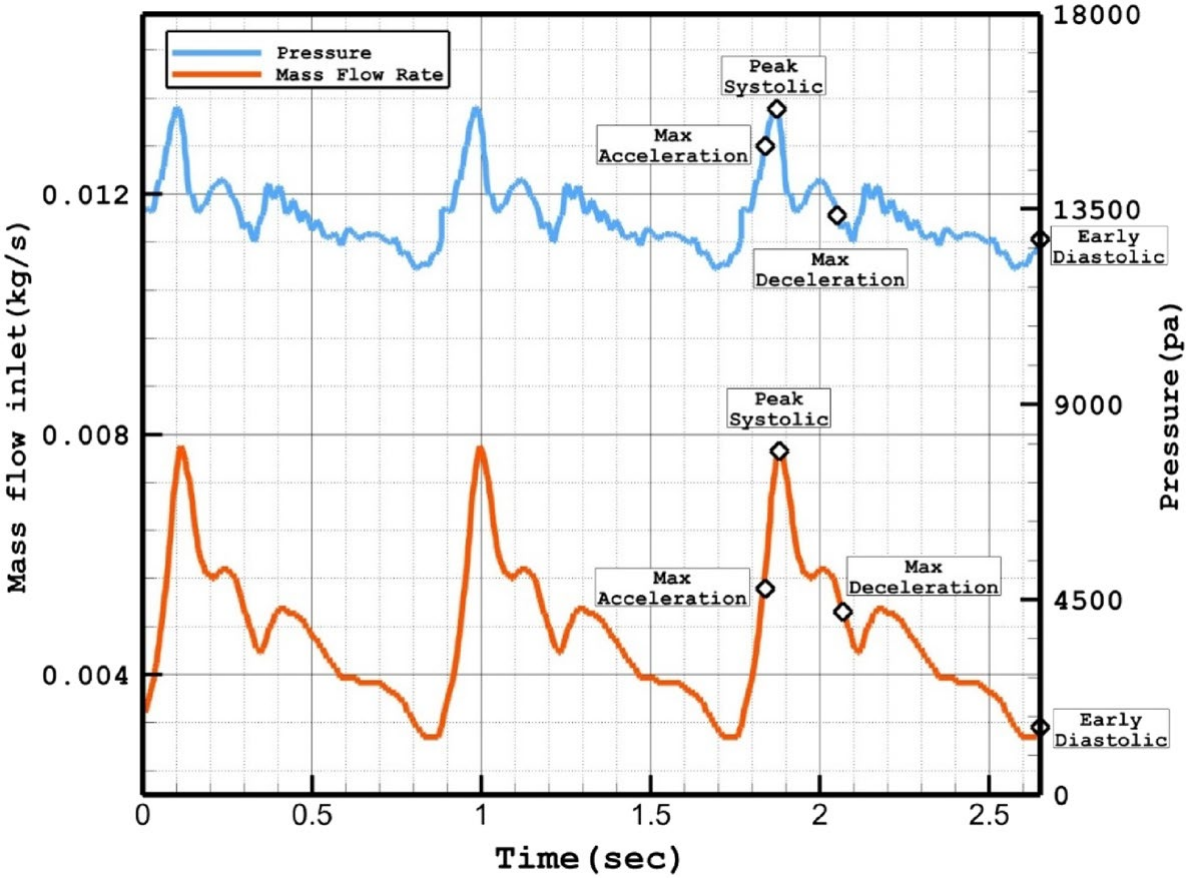
Applied mass flow rate and pressure profile at inlet and outlets ^1^.

The Casson model was used to estimate the viscosity of blood rheology^26^. Three cardiac cycles were investigated due to the unsteady characteristics of blood flow (Fig. 2), and the results of the last cycle were selected to ensure that the results were not dependent on initial transients^27,28^. The inlet and outlet were positioned far enough from the aneurysm sac to enable the velocity profile to progress appropriately. The inlet blood flow was applied via mass flow rate, while the outlet was a pressure outlet. The reported value in this study is for the peak systolic condition, which is of particular importance^29–31^.

## Results and discussion

### Blood viscosity effects

Due to the importance of the blood HCT on hemodynamics, four different HCTs are examined on a specific model of D which has a mean sac centreline among selected models. Table 2 presents the impacts of various HCTs on the blood characteristics at peak systolic.

**Table 2:** Details of obtained results on selected model (D) for different HCTs.

HCT	WSS Max Sac (pa)	WSS Mean Sac (pa)	WSS Min Sac (pa)	OSI Max Sac	OSI Mean Sac	MWA_Vel_interior (m/s)	Sac mean pressure (pa)	Sac mean Velocity (m/s)
0.33	140.5824	17.0533	0.287034	0.417075	0.024434	1.016822	19,136.06	0.524941
0.4	147.6993	18.39566	0.230072	0.375356	0.013998	0.987321	19,328.86	0.509628
0.45	152.1064	19.258	0.233781	0.330751	0.009701	0.974953	19,551.4	0.498876
0.5	157.6807	19.8118	0.368156	0.341078	0.007257	0.954	19,875.23	0.48059

**Figure 3:**
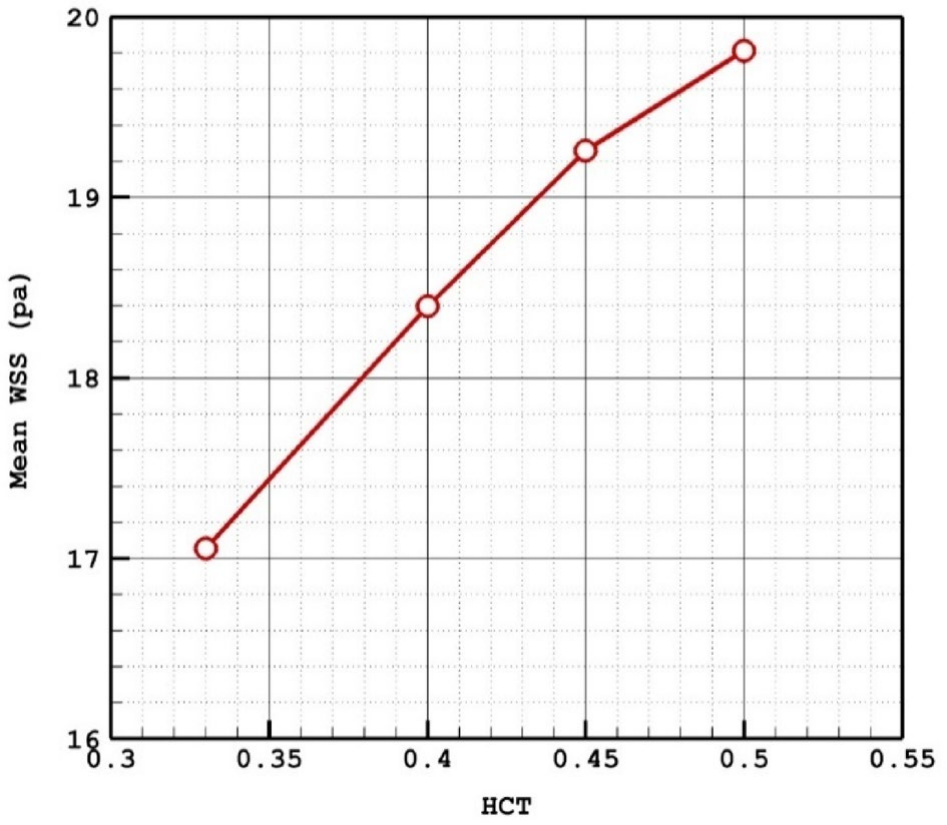
HCT effects on mean WSS.

**Figure 4:**
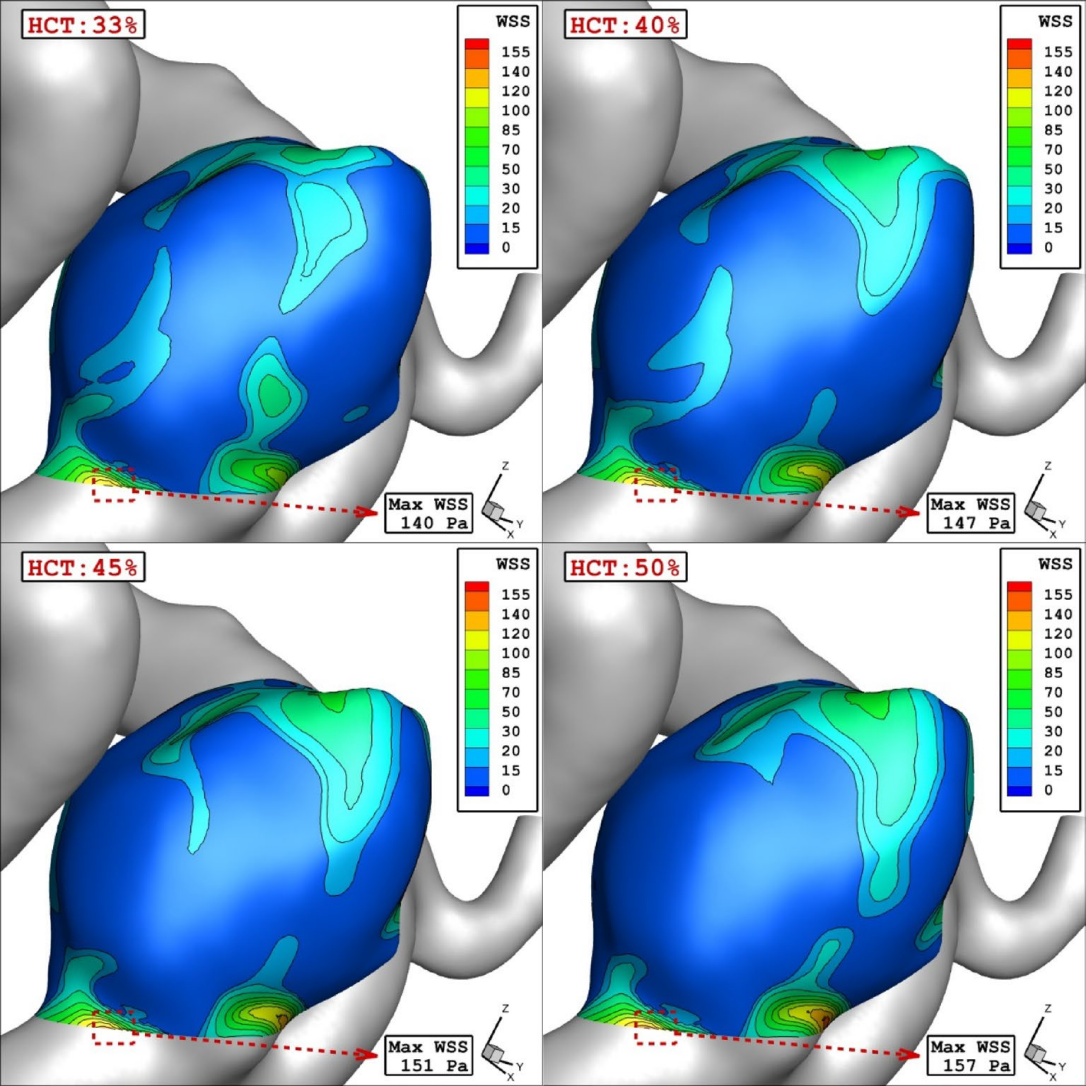
WSS contours in different HCTs.

The influence of HCT on the mean WSS on the selected aneurysm (model D) is demonstrated in Figs. 3 and 4. It is found that increasing the HCT directly raises the mean WSS on the chosen aneurysm. Figure 4 also confirms that the maximum value of WSS happens nearby the ostium section and it increased by rising the HCT.

**Figure 5:**
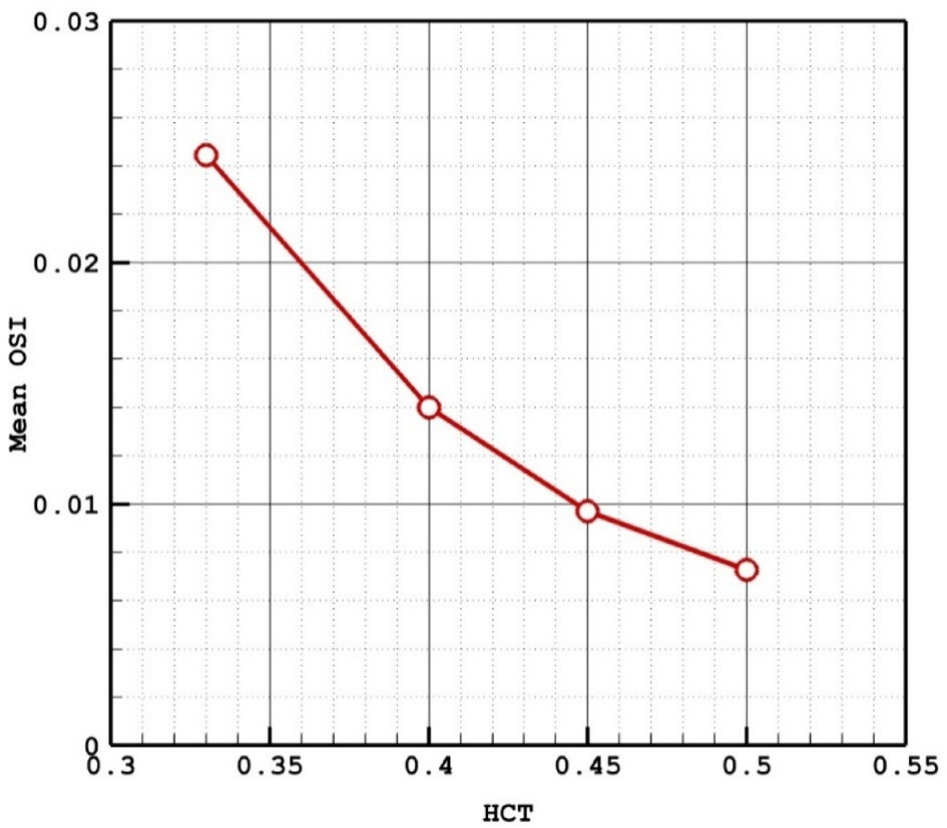
HCT effects on mean OSI.

**Figure 6:**
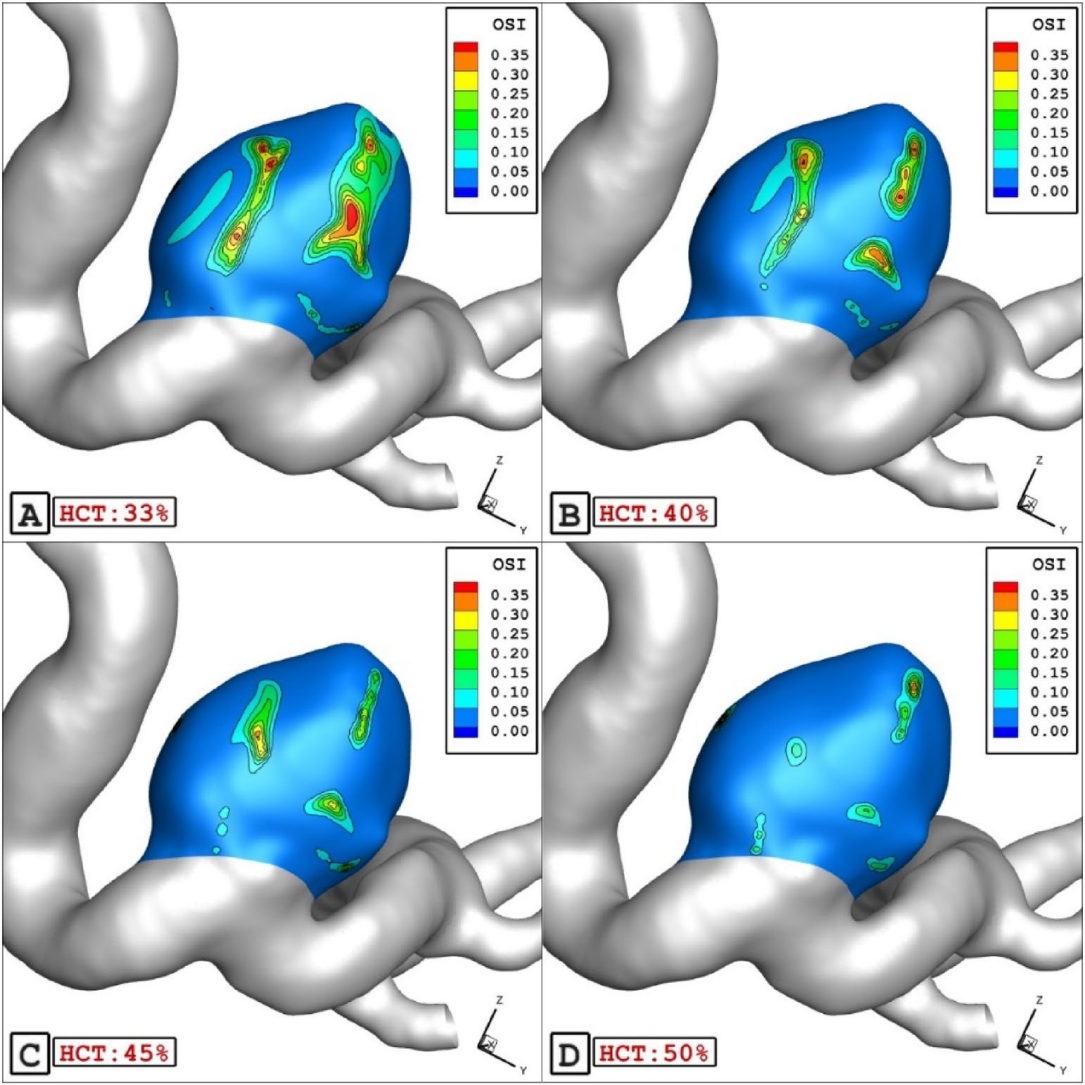
OSI contours in different HCTs.

The Fig. 5 plots the impacts of HCT on the OSI values at peak systolic instant. It is observed that the value of mean HCT decreases about 60% with 40% increase of HCT. Figure 6 also displays the variation of the OSI on the aneurysm wall and the variation of the OSI indicate that a high value of OSI occurs near the dome section.

**Figure 7:**
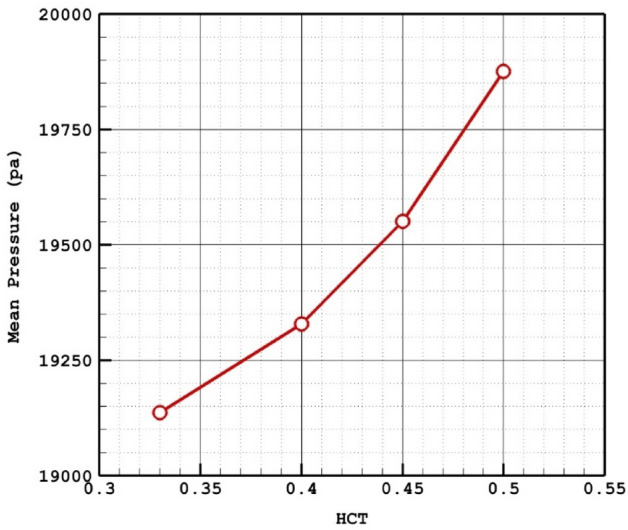
HCT effects on mean sac wall pressure.

**Figure 8:**
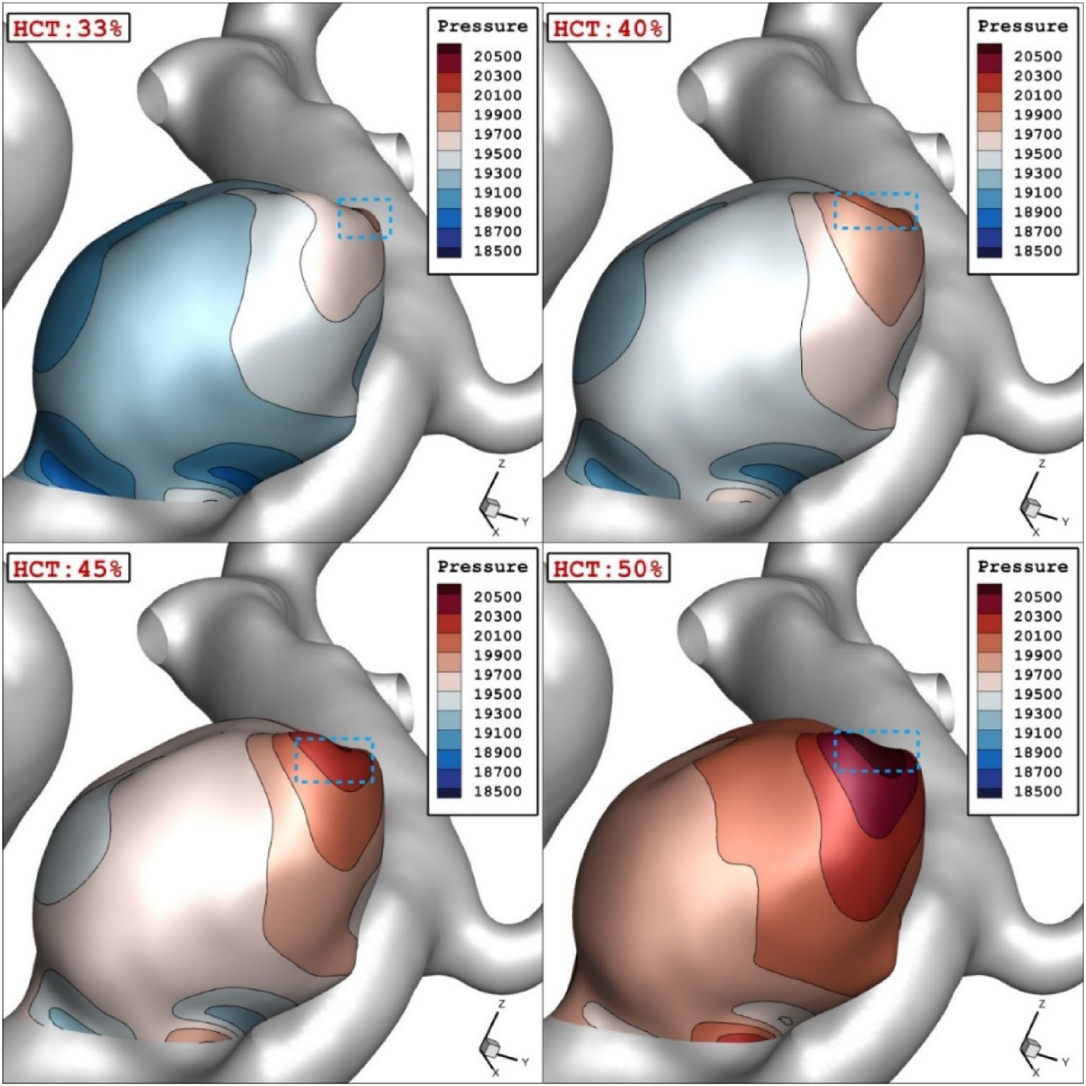
Wall Pressure contours in different HCTs.

The Figs. 7 and 8 demonstrate the impacts of the HCT on the mean pressure distribution on the aneurysm wall at peak systolic time instant. Since increasing the HCT results in high viscosity, the pressure on the aneurysm wall significantly increases and the high-pressure region happens exactly on the dome of the aneurysm as displayed in Fig. 8. In fact, the pressure distribution is the main reason for deformation and shape of the aneurysm.

**Figure 9:**
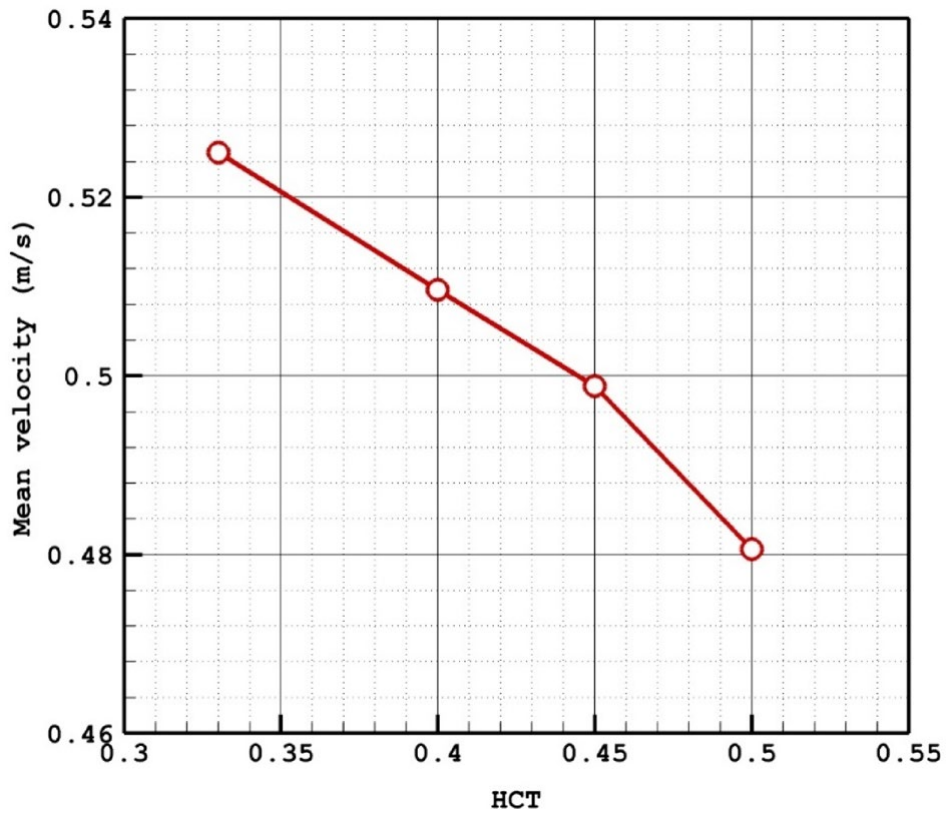
HCT effects on mean sac velocity.

**Figure 10:**
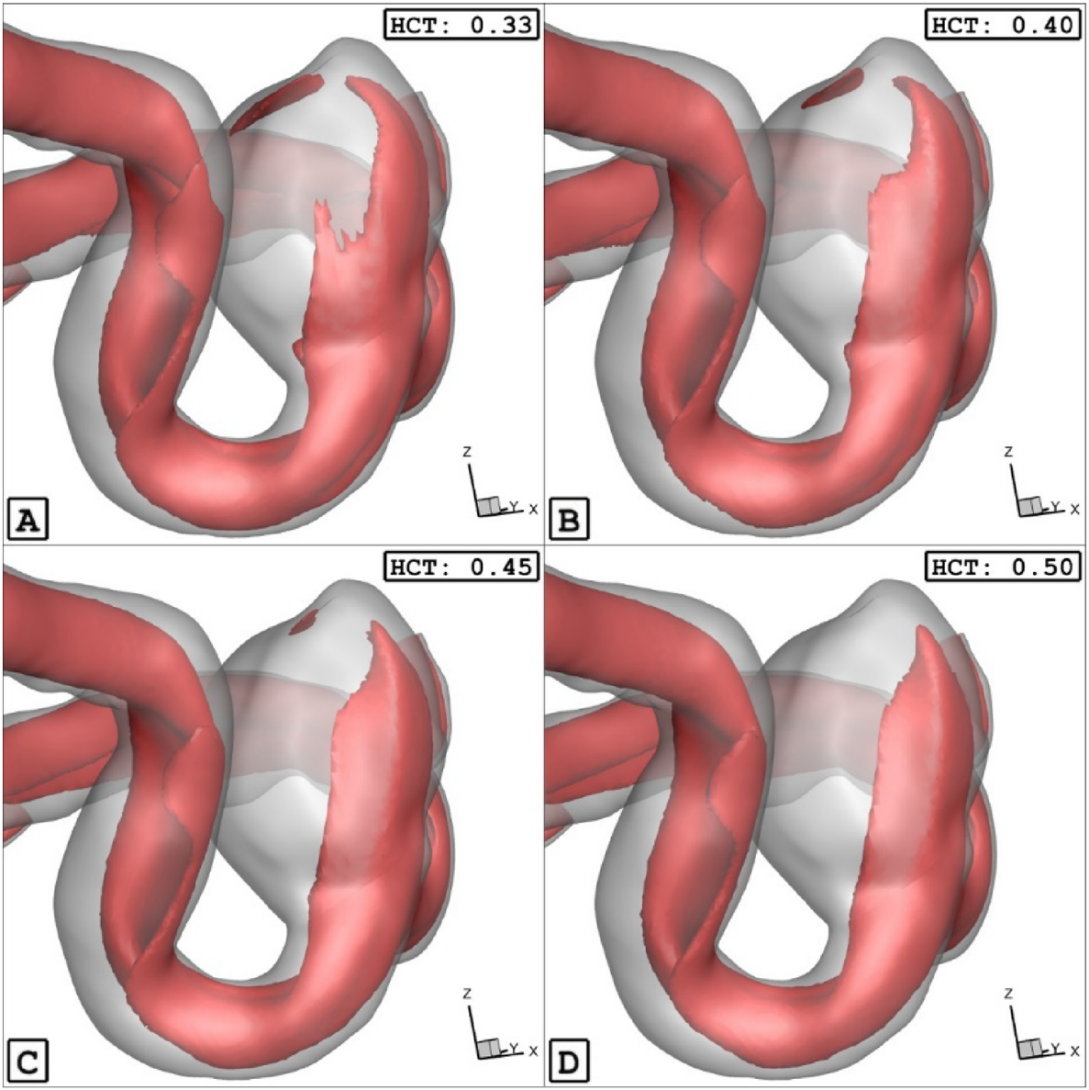
ISO surface velocity (1 m/sec) in different HCTs.

The Fig. 9 confirms that the increases of the HCt would result in lower mean velocity because of high viscosity. To notice the impacts of HCT, Fig. 10 illustrates the iso-velocity surface to compare the effects of the HCT on the blood hemodynamic inside the selected aneurysm at peak systolic instant. It is found that the HCt mainly impacts the blood feature inside the sac rather than the nearby wall.

### Coiling Porosity effects

**Figure 11:**
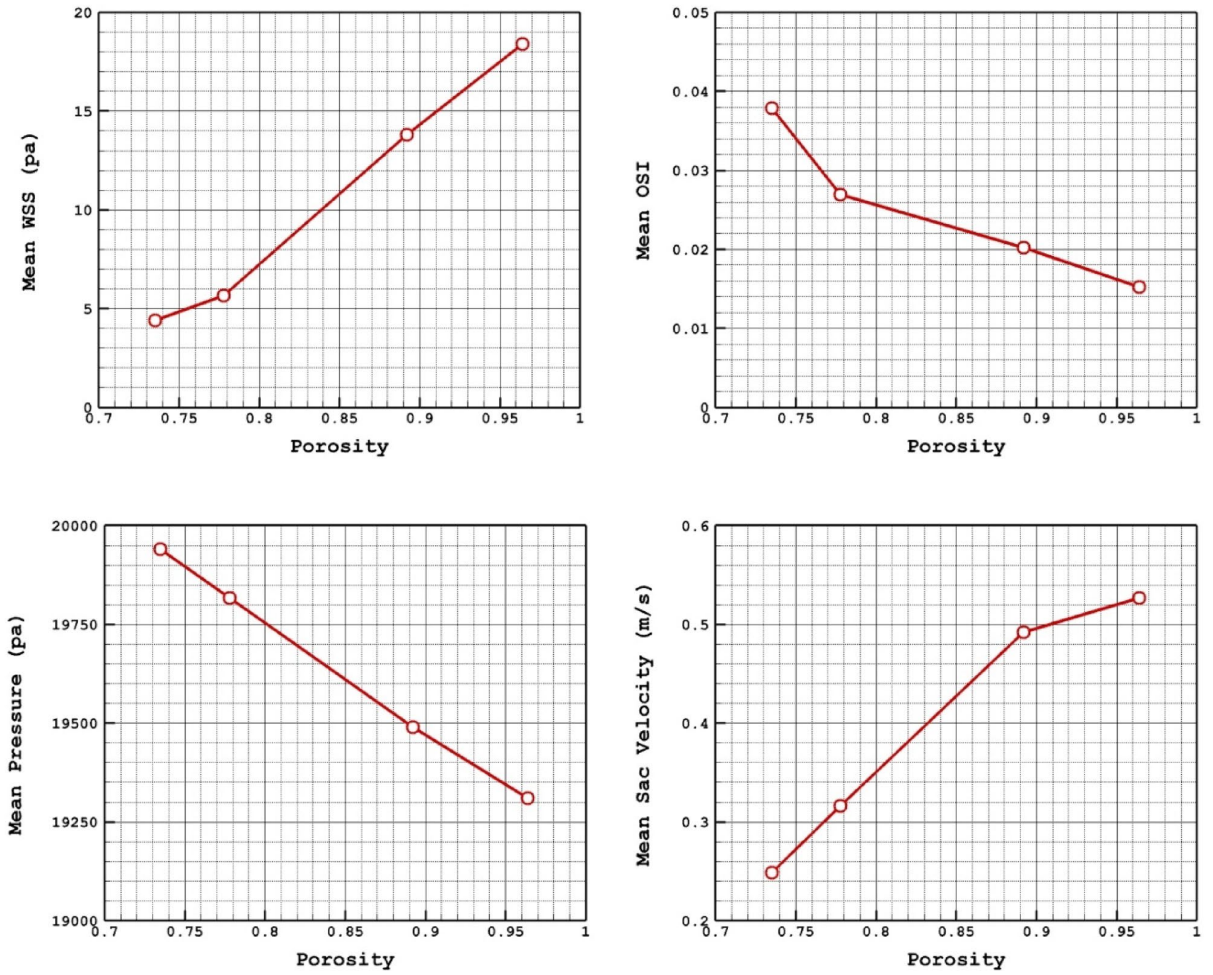
Porosity effects on mean values of WSS, OSI, sac pressure and sac velocity at peak systolic instant on the selected aneurysm (D).

The coiling technique is recognized as the main technique for the treatment of the saccular aneurysm. Table 3 presents the results of blood flow features in different coiling porosities. Figure 11 plots the porosity impacts on mean values of WSS, OSI, sac pressure, and sac velocity at peak systolic instant on the selected aneurysm (D).

**Table 3:** Details of obtained results on selected model (D) for different coiling porosity.

Permeability (m^2^ × 10^−8^)	Porosity	WSS Max Sac (pa)	WSS Mean Sac (pa)	WSS Min Sac (pa)	OSI Max Sac	OSI Mean Sac	MWA_Vel_interior (m/s)	Sac mean pressure (pa)	Sac mean velocity (m/s)
1.54548	0.735	131.835	4.393409	0.237484	0.447007	0.03781	1.107858	19,940.34	0.2484077
2.47361	0.778	137.5839	5.648677	0.534482	0.432753	0.026943	1.102091	19,816.03	0.3160101
13.7441	0.892	151.3244	13.81179	0.208602	0.443797	0.020252	1.009931	19,490.09	0.4916839
144.47	0.964	207.534	18.37318	0.233454	0.404799	0.015257	0.990007	19,309.5	0.5270406

**Figure 12:**
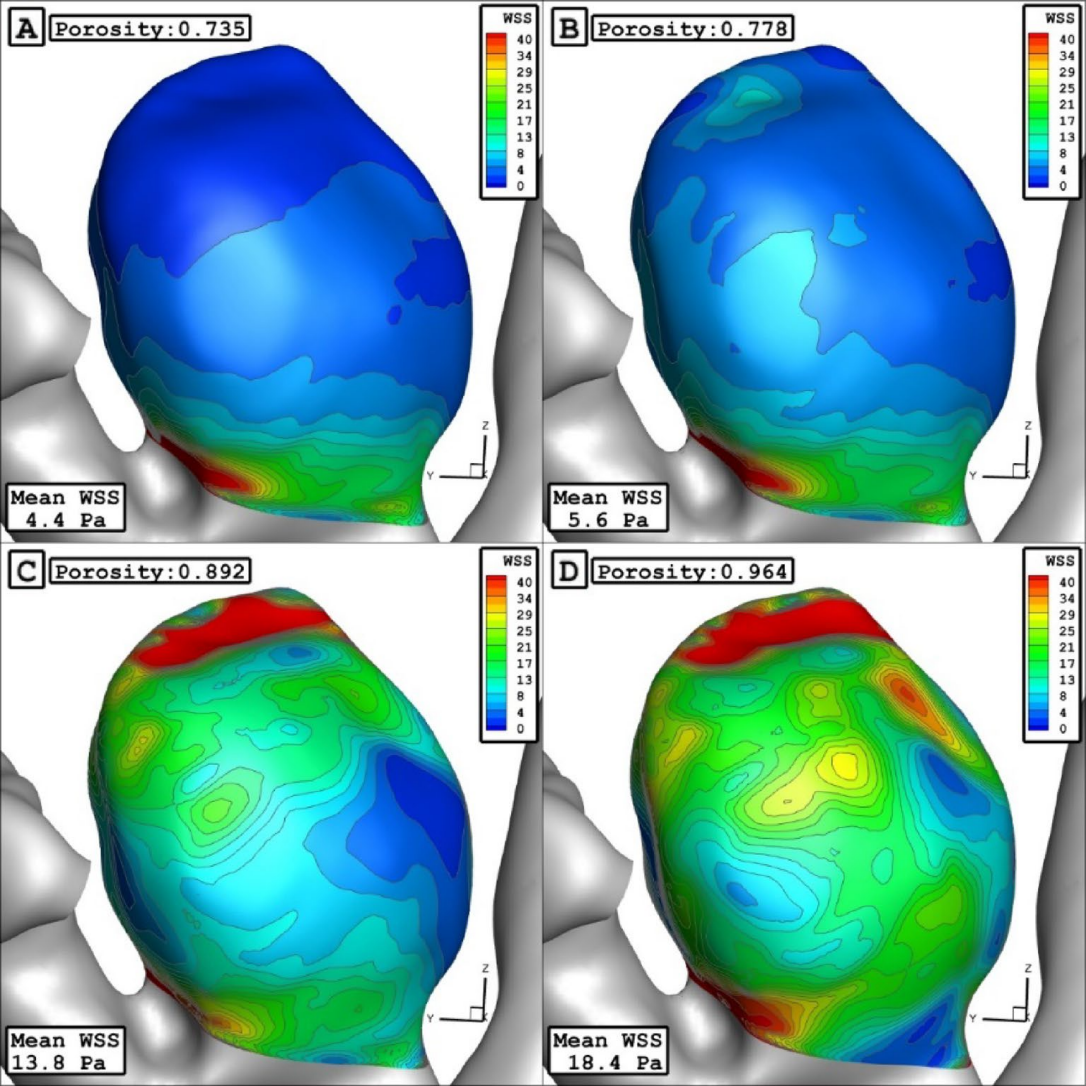
WSS contours in different porosities.

**Figure 13:**
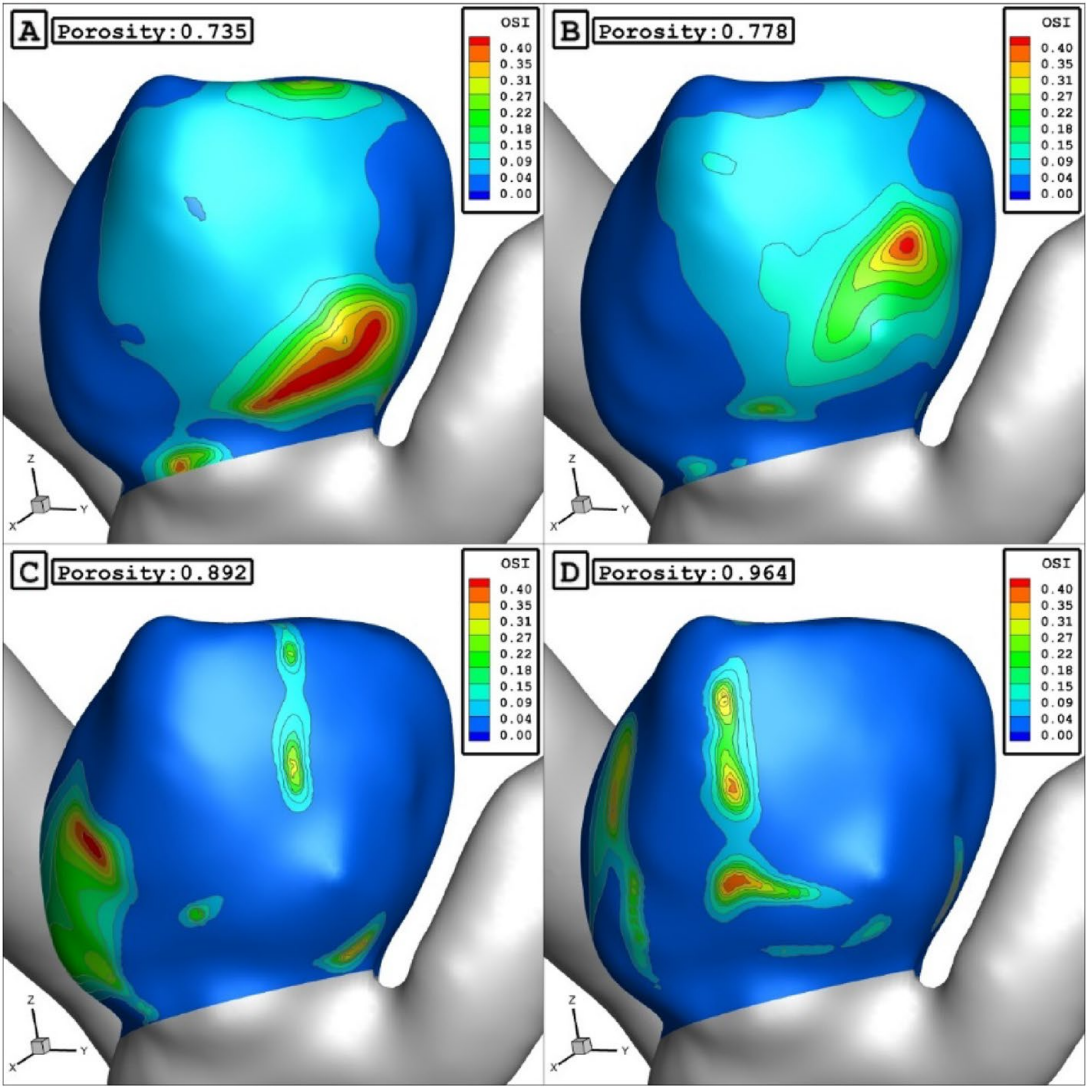
OSI contours in different porosities.

The Fig. 12 displays the effect of porosity on the WSS distributions. It is found that the maximum WSS happens nearby the dome in low porosity. As the porosity of coiling increases, it happens near the sac neck. As presented in Fig. 11, the increase of the porosity would increase the mean WSS and sac velocity while it decreases mean pressure and OSI inside the aneurysm. Figure 13 also confirms the distribution of OSI on the sac wall for different coiling porosities. It is observed that the high value of the OSI occurs in the middle of the domain far from the neck and dome.

**Figure 14:**
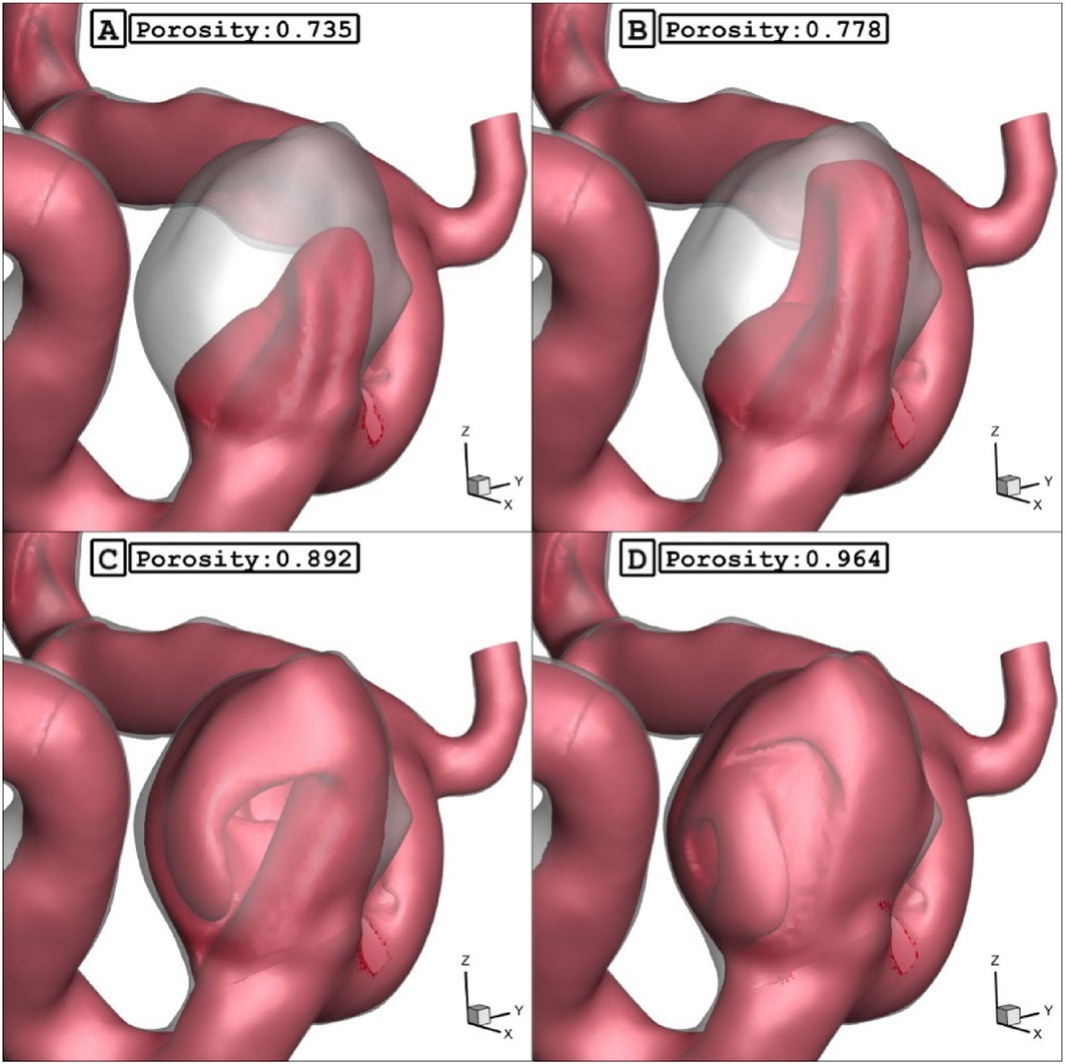
ISO surface velocity (0.3 m/sec) in different porosity.

**Figure 15:**
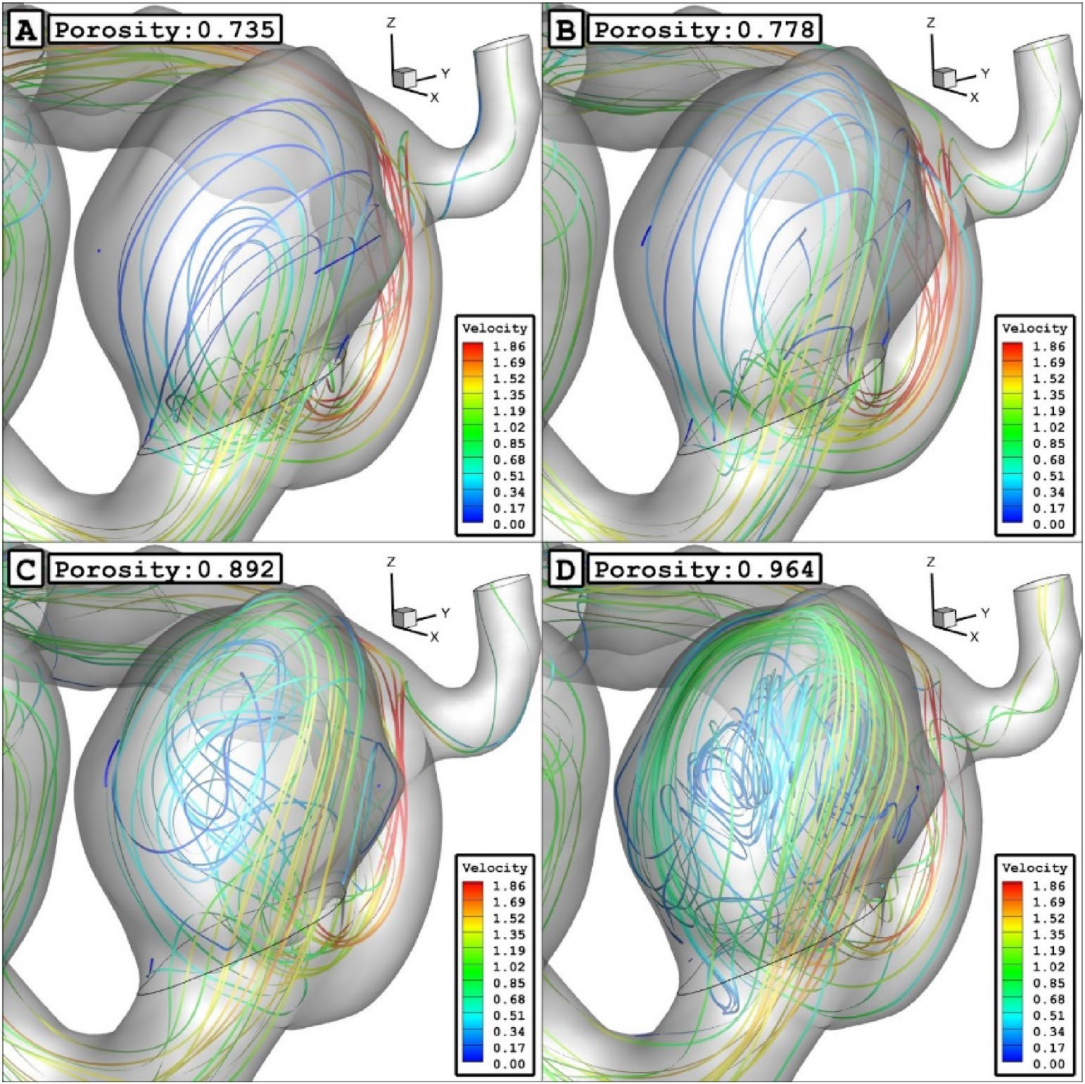
Streamlines in different porosity.

The Fig. 14 demonstrates the blood feature in presence of different coiling porosity. As noticed in the figure, the permeability of coiling decreases by increasing the coiling porosity. Therefore, the penetration of the blood stream inside the sac is limited as the porosity decreases inside the sac. As demonstrated in Fig. 15, the blood stream is highly limited when the permeability of coiling is increased (or porosity is decreased.)

## Conclusion

This study conducts thorough computational analyses to uncover the influence of blood hemodynamics on six distinct saccular ICAs. Specifically, the study extensively explores the effects of the sac centerline, which is a key geometric characteristic of the saccular aneurysm. The study compares the primary parameters of wall shear stress (WSS), oscillatory shear index (OSI), pressure, and velocity among these models with varying sac centerlines. Our work tries to discloses the effect of blood HCT on the risk of aneurysm rupture in one specific aneurysm in peak systolic time instant. Blood stream and feature are also compared for various viscosity of the blood flow. In addition, the influence of the coiling porosity on rupture risk of the aneurysm is fully investigated in the present work. According to our findings, increasing the HCT would increase the WSS and pressure value on the aneurysm wall while OSI and mean velocity decreases by high value of blood HCTs. Our results also show that the coiling technique could sufficiently decrease the risk of rupture since decreasing the coil porosity (increasing the coil permeability) would increase the OSI and pressure and decrease WSS and blood velocity inside the sac. It is observed that the value of mean HCT decreases about 60% with 40% increase of HCT.

## Data Availability

All data generated or analysed during this study are included in this published article.
